# Development and External Validation of a Nomogram for Predicting Survival in Patients With Stage IA Non-small Cell Lung Cancer ≤2 cm Undergoing Sublobectomy

**DOI:** 10.3389/fonc.2019.01385

**Published:** 2019-12-11

**Authors:** Yang Wo, Hongxia Yang, Yinling Zhang, Jinshan Wo

**Affiliations:** ^1^Thoracic Oncology Center, Affiliated Hospital of Qingdao University, Qingdao, China; ^2^Department of Oncology, The Second Affiliated Hospital of Qingdao University, Qingdao, China; ^3^Department of Cardiology, Affiliated Hospital of Qingdao University, Qingdao, China

**Keywords:** survival, lymph node, lung cancer, nomogram, surgery

## Abstract

**Background:** Postoperative prognosis of early stage non-small cell lung cancer (NSCLC) undergoing sublobectomy is heterogeneous. Therefore, we sought to construct a novel survival prediction model for stage IA NSCLC ≤2 cm undergoing sublobectomy.

**Methods:** Based on the data from the Surveillance, Epidemiology, and End Results (SEER) program, we successfully determined and incorporated independent prognostic markers to construct the nomogram. Internal validation of the constructed nomogram was conducted through 1,000 bootstrap resamples. The constructed nomogram was further subjected to external validation with an independent cohort of patients from two Chinese institutions. The performance of the survival prediction model was assessed by concordance index, calibration plots, and risk subgroup classification.

**Results:** A total of 3,238 patients from SEER registries (development cohort), as well as 769 patients from two Chinese institutions (validation cohort) was included. Gender, age, size, histologic type, grade, and examined lymph nodes count were identified as significant prognostic parameters. A novel nomogram was developed and externally validated. Concordance index of constructed nomogram was significantly better than that of the current TNM staging system. Calibration plots demonstrated an optimal consistency between the nomogram predicted and actual observed probability of survival. Survival curves of different risk subgroups within respective TNM stage demonstrated significant distinctions.

**Conclusion:** We developed and externally validated a survival prediction model for patients with stage IA NSCLC ≤2 cm undergoing sublobectomy. This novel nomogram outperforms the conventional TNM staging system and could help clinicians in postoperative surveillance and future clinical trial design.

## Introduction

The widespread application of advanced imaging technique for lung cancer screening has witnessed a dramatic increase in the early detection of non-small cell lung cancer (NSCLC) and renewed interests in assessing the optimal surgical procedures for NSCLC ≤2 cm (1, 2). Sublobar resection, which comprises segmentectomy and wedge resection, has been demonstrated by scholars to reach oncological outcome comparable to that of lobectomy in early NSCLC (3–6). Theoretical merits of limited resection include preserving lung functions and minimizing the peri-operative mortality risk for frail and aged patients with complex comorbidities, and the ability to perform further resections for second primary lung malignancy (5). The eighth edition of the American Joint Committee on Cancer (AJCC) TNM staging has been applied universally, in which stage IA NSCLC ≤2 cm are classified merely based on tumor size (T descriptors) (7). However, postoperative prognosis of cases with the same TNM stage is heterogeneous, and therefore the development of tools based on significant prognosticators such as demographic characters, pathologic features, and surgical procedures may contribute to personalized survival prediction (8).

Nomograms give numerical estimate of probabilities of specific clinical events and have been identified as reliable tools to visually assess the risks by incorporating vital factors for oncologic outcomes (8–10). In various types of malignancies, nomograms were proved to confer more precise survival prediction than the conventional TNM staging criteria. Patients with early stage NSCLC treated with sublobar resection are heterogeneous in distinct physical conditions and therapeutic strategies, which raises difficulty and uncertainty in survival prediction and risk group stratification. The most widely used AJCC TNM classification system stratifies stage IA NSCLC ≤2 cm merely based on tumor size and previously developed nomogram for NSCLC failed to consider tumor size or surgical strategy (11, 12), which necessitate the need to develop individualized survival prediction model for those patients. Therefore, we sought to develop a novel nomogram to quantify the postoperative prognosis of stage IA NSCLC ≤2 cm utilizing a cohort from population-based Surveillance, Epidemiology, and End Results (SEER) program, and externally validate it with a separate cohort of our institutions.

## Methods

### Study Cohorts

Data were retrieved from the SEER 18 registries (1975–2016) which covers nearly 28% of the US population. Detailed characteristics of patients with microscopically confirmed first primary NSCLC from 2004 to 2015 were retrospectively reviewed and our focus was narrowed to stage IA1-2 NSCLC undergoing sublobectomy. Also included are demographic characters, surgical procedures, tumor morphologies and vital status. Patients who received neoadjuvant radiotherapy or those with unknown included variables were excluded. Differentiation grade was reclassified as grade I (well-differentiated), grade II (moderately differentiated), and grade IIII/IV (poorly differentiated or undifferentiated). Histologic type was classified as adenocarcinoma (ADC), squamous cell carcinoma (SC), and others. A separate external validation cohort, which consisted of 769 eligible cases diagnosed between 2007 and 2012 in two institutions in Qingdao (the Affiliated Hospital of Qingdao University and the Second Affiliated Hospital of Qingdao University), was constructed to assess the generalizability of the prognostic model. Our institutional review board approved this study.

### Statistical Analysis

Lung cancer-specific survival (LCSS), defined as the interval from medical diagnoses to lung cancer-related death, was the end point parameter and was assessed with Kaplan-Meier analyses and log-rank tests. Nomogram was constructed based on independent prognostic variables identified by multivariate Cox regression analyses. The constructed nomogram was further subjected to external validation with an independent cohort of patients from two Chinese institutions, while internal validation of the constructed nomogram was conducted through 1,000 bootstrap resamples. The discriminative performance of prediction models was assessed with concordance index (C-index). The calibration for 3 and 5-year LCSS (consistency between the actual and predicted LCSS) was evaluated by visual assessment of generated calibration plots. To better clarify the independent discrimination performance of our constructed nomogram, we therefore reclassified patients into three risk groups according to total risk scores in development cohort. The optimal cut-point of total risk scores was identified by X-tile software based on minimal *P-*value approach (13).

Statistical analyses were conducted on SPSS 22.0 (IBM, Armonk, NY) and R 3.5.3 (R foundation, Vienna, Austria) with *rms* and *survival* packages. All tests were two-sided, and *P* < 0.05 was statistically significant.

## Results

### Patient Characteristics

A total of 3,238 patients from SEER registries (development cohort), as well as 769 patients from two Chinese institutions (validation cohort), was identified based on the inclusion criteria. The median (interquartile range) follow-up duration was 43 (23–72) month and 74 (40–87) month for the development and validation cohorts, respectively. Most of patients were aged at least 70 years, and the proportion of female was greater than that of male in both cohorts. Nearly 80% of cases underwent wedge resection. With regard to lymph nodes (LNs) examination, nearly 40% patients in the development cohort had no LNs evaluated, while only 18.2% of those in the validation cohort did not. Baseline characteristics are listed in Table 1.

**Table 1:** Patients characteristics.

**Variable**	**Development cohort**		**Validation cohort**	
	**No. patients**	**%**	**No. patients**	**%**
Age groups, y				
<60	589	18.2	149	19.4
60–70	1,183	36.5	264	34.3
>70	1,466	45.3	356	46.3
Histologic type				
Adenocarcinoma	2,063	63.7	493	64.1
Squamous cell carcinoma	760	23.5	196	25.5
Others	415	12.8	80	10.4
Sex				
Male	1,318	40.7	312	40.6
Female	1,920	59.3	457	59.4
Race				
White	2,823	87.2		
Black	255	7.9		
Other	160	4.9	769	100.0
Marital status				
Married	1,829	56.5	415	54.0
Single	357	11.0	74	9.6
Other	1,052	32.5	280	36.4
Grade				
I	986	30.5	215	28.0
II	1,416	43.7	357	46.4
III/IV	836	25.8	197	25.6
Tumor size, cm				
≤1	804	24.8	169	22.0
1.1–2	2,434	75.2	600	78.0
Surgery				
Wedge resection	2,587	79.9	620	80.6
Segmentectomy	651	20.1	149	19.4
ELNs				
0	1,393	43.0	140	18.2
1–5	1,248	38.5	298	38.8
6–10	359	11.1	211	27.4
>10	238	7.4	120	15.6

*ELNs, examined lymph nodes*.

### Identification of Independent Prognostic Factors for the Development Cohort

**Figure 1:**
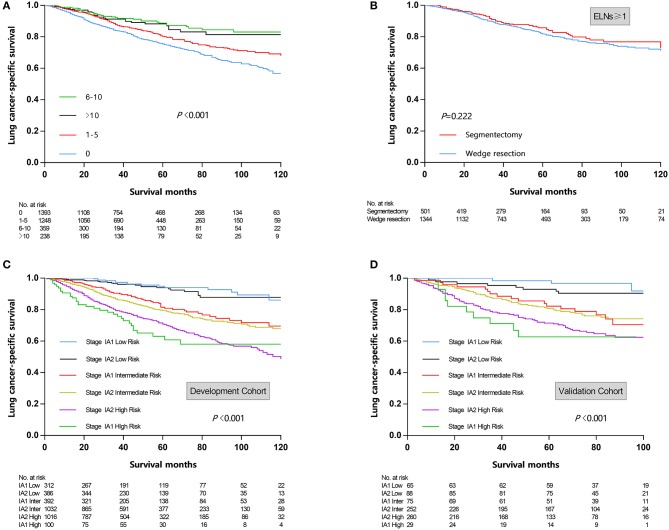
**(A)** Survival curves stratified by examined lymph nodes count. **(B)** Survival curves stratified by surgical procedure (wedge resection vs. segmentectomy) in patients undergoing lymph nodes examination. **(C)** Risk group stratification within respective TNM stage in development cohort. **(D)** Risk group stratification within respective TNM stage in validation cohort. ELNs, examined lymph nodes.

In univariate Cox regression analysis (Table 2), the clinicopathologic and demographic factors that demonstrated a significant association with LCSS were gender (*P* < 0.001), age (*P* < 0.001), marital status (*P* = 0.018), size (*P* < 0.001), histologic type (*P* = 0.003), differentiation grade (*P* < 0.001), surgical procedure (*P* = 0.021), and examined lymph nodes (ELNs) count (*P* < 0.001). All of the above identified prognostic factors were incorporated into the multivariate Cox regression models, which indicated that gender (*P* = 0.001), age (*P* < 0.001), size (*P* < 0.001), histologic type (*P* = 0.034), grade (*P* < 0.001), and ELNs (*P* < 0.001) were independent prognostic factors for LCSS, while marital status (*P* = 0.161), and surgical procedure (*P* = 0.124) were not (Table 2). On stratification of ELNs, a significant positive trend between the retrieved LNs count and LCSS was identified, and the maximal survival benefit was achieved with 6–10 ELNs; however, examination of more than 10 LNs did not confer superior LCSS (Figure 1A). Furthermore, it is worth noting that wedge resection was no longer correlated with decreased survival in patients with at least one examined LNs (Figure 1B).

**Table 2:** Identification of independent prognostic factors for the development cohort.

**Variable**	**Univariate**		**Multivariate**	
	**Hazard Ratio (95% CI)**	***P-*value**	**Hazard Ratio (95% CI)**	***P*-value**
Age groups, y		<0.001		<0.001
<60	Reference		Reference	
60–70	1.482 (1.133–1.938)	0.004	1.315 (1.003–1.725)	0.048
>70	2.074 (1.607–2.677)	<0.001	1.702 (1.309–2.212)	<0.001
Histologic type		0.003		0.034
Adenocarcinoma	Reference		Reference	
Squamous cell carcinoma	1.315 (1.096–1.576)	0.001	0.870 (0.720–1.051)	0.113
Others	0.811 (0.620–1.060)	0.126	0.728 (0.553–0.960)	0.015
Sex		<0.001		0.001
Male	Reference		Reference	
Female	0.719 (0.613–0.843)	<0.001	0.760 (0.643–0.897)	0.001
Race		0.118		
White	Reference			
Black	0.903 (0.668–1.220)	0.507		
Other	0.628 (0.398–0.993)	0.047		
Marital status		0.018		0.161
Married	Reference		Reference	
Single	0.822 (0.615–1.099)	0.187	0.849 (0.633–1.138)	0.274
Other	1.200 (1.014–1.421)	0.034	1.123 (0.940–1.342)	0.202
Grade		<0.001		<0.001
I	Reference		Reference	
II	2.659 (2.085–3.391)	<0.001	2.411 (1.879–3.092)	<0.001
III/IV	3.424 (2.663–4.404)	<0.001	3.226 (2.486–4.187)	<0.001
Tumor size, cm		<0.001		<0.001
≤1	Reference		Reference	
1.1–2	1.601 (1.300–1.971)	<0.001	1.441 (1.169–1.777)	<0.001
Surgery		0.021		0.124
Wedge resection	Reference		Reference	
Segmentectomy	0.777 (0.627–0.963)	0.021	0.842 (0.677–1.048)	0.124
ELNs		<0.001		<0.001
0	Reference		Reference	
1–5	0.725 (0.611–0.860)	<0.001	0.755 (0.634–0.899)	0.002
6–10	0.419 (0.298–0.589)	<0.001	0.477 (0.339–0.672)	<0.001
>10	0.514 (0.351–0.751)	0.001	0.568 (0.387–0.833)	0.004

*CI, confidence interval; ELNs, examined lymph nodes*.

### Nomogram Construction

**Figure 2:**
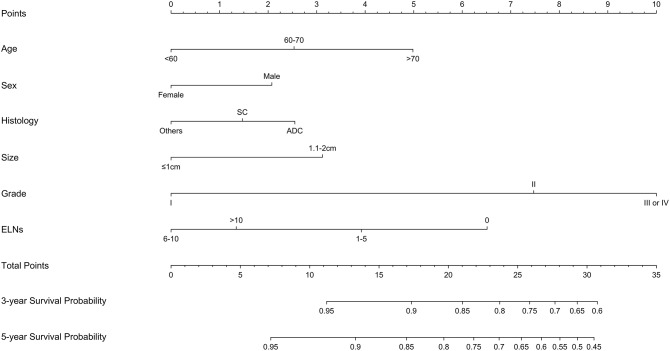
Constructed nomogram for predicting survival in patients with stage IA non-small cell lung cancer ≤2 cm undergoing sublobectomy. SC, squamous cell carcinoma; ADC, adenocarcinoma; ELNs, examined lymph nodes.

A nomogram was established based on the independent prognostic factors derived from multivariate analysis (Figure 2). The nomogram elucidated differentiation grade and ELNs count as sharing the greatest contribution for LCSS, while the gender and histologic type conferred a moderate impact on LCSS. Each descriptor or subtype of incorporated parameters was assigned a risk score on the point scale. Then, the total risk scores of individuals, ranging from 0 to 35, was calculated. Finally, we were able to visually estimate the 3- and 5-year LCSS by drawing a straight line from total points scale down to the 3- and 5-year survival probability scales.

### Calibration and Validation

**Figure 3:**
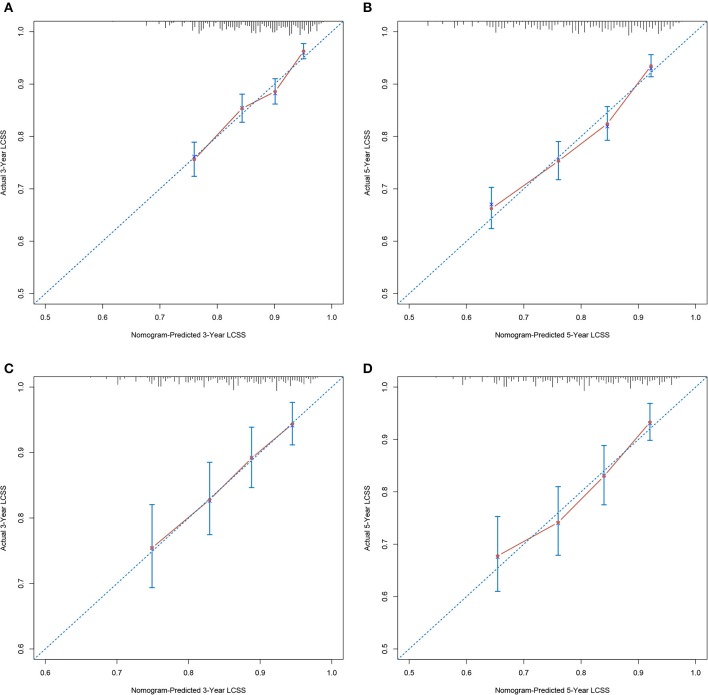
Calibration plots demonstrating the consistency between the nomogram predicted and actual observed 3- and 5-year LCSS in **(A,B)** development cohort and **(C,D)** validation cohort. LCSS, lung cancer-specific survival.

Calibration plots of the development cohort (Figures 3A,B) and the external validation cohort (Figures 3C,D) demonstrated an optimal consistency between the nomogram predicted and actual observed 3- and 5-year LCSS. In the development cohort, the C-index for the constructed nomogram to predict LCSS [0.674; 95% confidence interval (CI), 0.652–0.696] outperformed the TNM staging criteria (0.542; 95% CI, 0.525–0.559; *P* < 0.001). In the external validation cohort, the C-index of the novel nomogram (0.666; 95% CI, 0.628–0.704) was also greater than the conventional TNM-based model (0.538; 95% CI, 0.510–0.566; *P* = 0.011).

### Stratification of Risk Groups

According to cut-point analysis via X-tile program, patients were divided into three risk groups: low risk (score 0–13.9), intermediate risk (score 14.0–21.9), and high risk (score 22.0–35.0). When the above cut-points were applied to the development cohort, each risk subgroups demonstrated significant distinctions between survival curves within the same T descriptor, while patients within the same risk group shared similar survival probability despite the T descriptor, which indicated that the novel nomogram could provide more precise survival prediction than the conventional TNM-based model (Figure 1C). In the external validation cohort, a similar trend was observed (Figure 1D).

## Discussion

Despite various previously developed predictive models (8, 10, 14–16), a nomogram for resected small sized (≤2 cm) NSCLC has not been established. Therefore, we aimed to construct a novel prognostic model for stage IA NSCLC ≤2 cm undergoing sublobectomy. It was developed from a population-based cohort from US and externally validated by a separate cohort from two Chinese institutions. The capacity to incorporate multiple clinicopathologic parameters in a user-friendly model allowed nomogram to more precisely predict prognosis than conventional TNM-based models, which may benefit postoperative surveillance, clinical trial design, and treatment decision making.

Sublobar resection, which comprises segmentectomy and wedge resection, has been demonstrated by scholars to reach oncological outcome comparable to that of lobectomy in stage IA NSCLC (3–6). Although it is widely accepted that segmentectomy is a better oncologic surgery than wedge resection, only 20% of cases underwent segmentectomy in this study. Characterized by less technical complexity, shorter operation duration, and a greater chance of being completed via minimal invasive approach, wedge resection was performed for a great proportion of early stage NSCLC and has stimulated continued research concerning the oncologic outcome of these two operations (17–20). In this study, segmentectomy was not correlated with improved prognosis after adjusting for potential confounders. Interestingly, segmentectomy failed to confer additional survival advantage over wedge resection in patients who underwent LNs examination, which questioned the necessity of conducting technically complex segmentectomy if wedge resection with thorough LNs examination might be adequate. However, our current study failed to clarify this question and future prospective studies regarding this issue were encouraged.

The standard treatment modality of curative surgery for NSCLC involved systematic mediastinal LNs dissection (21). The prognostic significance of adequate LNs dissection was widely held for precise nodal staging and identifying adjuvant therapy candidates (22). However, this notion was challenged by ACOSOG Z0030 trial, which showed that systematic LNs dissection no longer improved oncologic outcome for early stage NSCLC if thorough LNs sampling indicated node negative disease (23). Considering the less parenchymal resection nature of sublobectomy is correlated with reduced possibility of sampling LNs and treating microscopic disseminated disease, the significance of adequate LNs examination during sublobectomy needs to be emphasized, and not surprisingly, the extent of LNs dissection was rigorously controlled in randomized trials comparing lobectomy and sublobectomy (24, 25). Unfortunately, a great number of patients did not receive LNs dissection during sublobectomy. Our study revealed that LNs examination was performed in only 57% of patients in US SEER cohort and 81.8% of patients in the Chinese cohort, indicating a significant mismatch between what is exactly being operated and what is considered to be crucial. Of interest in aforementioned results is that the maximal survival benefit was achieved with 6–10 ELNs and examination of more than 10 LNs did not confer superior LCSS. However, Samayoa and colleagues concluded that examination of at least 10 LNs reduced mortality risk and improved staging accuracy for T1-2N0M0 NSCLC (26). It is worth noting that the study conducted by Samayoa and colleagues did not stratify patients by tumor size and T descriptor. Considering patients with smaller tumors and earlier T-stage had a lower incidence of nodal metastasis (27), we therefore speculated that a less extensive LNs examination allowed sufficient staging accuracy for stage IA ≤2 cm lesions. While the purpose of this research is to demonstrate not the “optimal number” of LNs which should be examined based on specific clinical contexts, but that LNs examination significantly improved LCSS and should be encouraged for stage IA NSCLC ≤2 cm undergoing sublobectomy.

Tumor differentiation grade was identified as an effective parameter in evaluating the aggressiveness of tumors (28, 29). Our study demonstrated that the degree of differentiation had the greatest impact on LCSS. While it is encouraging to hear that the differentiation grade is now incorporated into the pathologic staging for early esophageal cancer (30), this parameter has not been included in the TNM staging criteria for lung cancer. Considering differentiation grade could potentially guide surgical procedure and predict survival, we strongly recommend the inclusion of differentiation grade in the forthcoming TNM classification.

Validation of the prediction model is crucial in determining the generalizability and preventing overfitting (31). The calibration plots in this study demonstrated an optimal consistency between the nomogram predicted and actual observed 3- and 5-year LCSS in both the SEER cohort and the Chinese cohort, which ensured the reliability and generalizability of our nomogram. In the current TNM-based model, the only parameter that could be adopted to subdivide stage IA NSCLC ≤2 cm is T descriptor (T1a vs. T1b); however, the discriminative capacity of T descriptor alone for LCSS was far from satisfactory. By incorporating six independent prognosticators, the novel prediction model performed significantly better than current TNM-based model. Moreover, we stratified patients into three risk subgroups. Interestingly, survival curves of different risk subgroups within the same T descriptor demonstrated significant distinctions, while patients within the same risk group shared similar survival probability despite the T descriptor, which further confirmed that the novel nomogram outperformed the conventional TNM-based model.

Our study also has several limitations. First, it was limited by the retrospective nature, and therefore inherent bias could not be completely dispelled. Second, we failed to incorporate other valuable prognostic parameters. The SEER registry lacks information regarding the pulmonary function, postoperative complication, updated adenocarcinoma classification, gene mutation, ^18^F-fluorodeoxyglucose positron emission tomography (PET) imaging, and proteomics analysis. In recent years, epidermal growth factor receptor-tyrosine kinase inhibitors (EGFR-TKIs) therapy and Anti-PD1 therapy are widely used for advanced NSCLC (32–36). Our study cohorts date back from 2004 to 2015, when targeted therapy and immunotherapy were not well-established, and molecular test was not routinely performed. Nowadays, EGFR-TKIs therapy and Anti-PD1 therapy remain controversial for very early stage NSCLC and are not usually recommended for resected early stage NSCLC in most of the guidelines. Although incorporating genetic or molecular information could perfectly improve the predictive value of this nomogram and provide more insights into this topic, our nomogram incorporates several valuable clinically available variables and is cheaper than molecular tests, making it a more economical and practical option for survival prediction. PET characteristics of these tumors, such as baseline metabolic tumor volume are innovative and valuable prognostic factors for NSCLC (37–39). Incorporating these characteristics would definitely make the nomogram more precise and meaningful. However, PET-CT scan is usually recommended for patients with advanced stage disease or with suspected distant metastasis in our institutions. Our study mainly focused on very early stage NSCLC (Stage IA1-IA2) and chest thin-section CT is routinely performed for small pulmonary nodules. Further efforts on prospective study results collection, broader geographic recruitment, and incorporation of aforementioned factors are encouraged to improve this nomogram. Additionally, although the established nomogram could stratify patients into different risk subgroups and precisely predict oncologic outcome, its application in guiding adjuvant therapy for stage IA NSCLC ≤2 cm has not been clarified. Future prospective studies are warranted to validate its performance in identifying potential adjuvant therapy candidates.

In conclusion, we developed and externally validated a nomogram for patients with stage IA NSCLC ≤2 cm undergoing sublobectomy. This novel nomogram, which estimates the 3- and 5-year survival of NSCLC individuals, outperforms the conventional TNM staging system and could benefit postoperative surveillance, future clinical trial design, and precision medicine development.

## Data Availability Statement

The datasets generated for this study are available on request to the corresponding author.

## Author Contributions

YW and JW proposed the conception and design of this research and analyzed and interpreted the data. HY and YZ developed methodology. YW and HY collected data and performed preprocessing. YW, JW, and HY were major contributors in writing the manuscript. All authors read and approved the final manuscript.

### Conflict of Interest

The authors declare that the research was conducted in the absence of any commercial or financial relationships that could be construed as a potential conflict of interest.
